# Text-Book of Physiology

**Published:** 1873-09

**Authors:** 


*Text-Book of Physiology, General, Special, and Practical.* By John Hughes Bennett, M. D., F. R. S. E., etc., with twenty-one Photo-Lithographic Plates. Philadelphia: J. B. Lippincott & Co., 1873. Buffalo: H. H. Otis.

This work embraces in a small space all that is of importance to a text book of Physiology. The subject matter is arranged under the following heads: I. General Physiology including Chemistry of the Tissues, General Histology, Physical and Vital Properties of the Tissues, On Life and Vitality. II. Special Physiology comprising Nutrition, Innervation, Reproduction and Death. Part III. Practical Physiology is divided into three sections including Practical, Chemical, Histological and Experimental Physiology.

The illustrations are placed at the end of the book and although sufficient in number it seems that their clearness has in a great measure been sacrificed to space.

The subjects are treated to as great an extent as is generally desirable in a text book, and the ideas advanced are in accordance with the accepted opinions of the day. A careful perusal of the work will well repay any student of Physiology.

